# Supplementary material for: Multiplex serology reveals age-specific immunodynamics of endemic respiratory pathogens in the wake of the COVID-19 pandemic
Source: medRxiv. 2025 Mar 27:2025.03.26.25324738. Preprint. [Version 1] doi: 10.1101/2025.03.26.25324738 (PMC12478342; doi:10.1101/2025.03.26.25324738)
Supplement: Supplement 1 [file media-1.pdf]

Supplementary Material

I. Seattle serology: Number of study participants by pathogen and age group and p-values results for Kolmogorov-Smirnov tests by pathogen and age group comparing average antibody concentration levels in the 2020 baseline year to 2021 and 2022.

Counts by pathogen and age group			
Age group	2020 (N)	2021 (N)	2022 (N)
<1	40	42	19
1-2	70	78	61
3-4	47	69	57
5-10	166	196	154
18-49	–	128	99
50-64	–	147	134
65+	–	141	153

Kolmogorov-Smirnov p-value results by pathogen and group comparing antibody concentration levels in 2021 to 2020 baseline. * denotes a significant difference between antibody concentration levels by year (p-value < .05) and ** denotes a significant difference after adjustment for multiple comparisons (p-value < .00125).				
**Pathogen**	**<1 yo**	**1-2 yo**	**3-4 yo**	**5-10 yo**
CoV-HKU1 Spike	0.1850	0.0006**	0.3516	0.4177
CoV-OC43 Spike	0.3068	0.0013*	0.0558	0.0644
Flu B Brisbane	0.1570	0.0101*	0.4138	0.0744
Flu-B-Phuket	0.2657	0.0213*	0.6430	0.0732
Flu-H1-Michigan	0.0292*	0.0120*	0.0587	0.5285
Flu-H3-HongKong	0.1007	0.0051*	0.0554	0.0595

HCoV NL63 Spike	0.0132*	0.0015*	0.0009**	0.0050*
HCoV-229E Spike	0.0130*	0.0008**	0.0013*	0.1574
RSV Pre-F	0.2251	0.0007**	0.0007*	0.0035*
SARS-CoV-2 N	0.3231	0.2574	0.3911	0.5299
SARS-CoV-2 RBD	0.2194	0.3259	0.2312	0.0586
SARS-CoV-2 Spike	0.1197	0.1934	0.2230	0.0256*

Kolmogorov-Smirnov results by pathogen and group comparing concentration levels in 2022 to 2020 baseline. * denotes significant p-value < .05 and ** denotes significant p-value after adjustment for multiple comparisons. .00125

**Pathogen**	**<1 yo**	**1-2 yo**	**3-4 yo**	**5-10 yo**
CoV-HKU1 Spike	0.137	0.064	0.125	0.057
CoV-OC43 Spike	0.475	0.372	0.235	0.200
Flu B Brisbane	0.020*	0.056	0.380	0.008*
Flu-B-Phuket	0.043*	0.107	0.834	0.002*
Flu-H1-Michigan	0.058	0.010*	0.045*	0.067
Flu-H3-HongKong	0.048*	0.143	0.001**	0.040*
HCoV NL63 Spike	0.252	0.302	0.043*	0.609
HCoV-229E Spike	0.161	0.122	0.530	0.547
RSV Pre-F	0.034*	0.114	0.004*	0.317
SARS-CoV-2 N	0.003*	0.000**	0.000**	0.000**
SARS-CoV-2 RBD	0.000**	0.000**	0.000**	0.000**

SARS-CoV-2 Spike	0.000**	0.000**	0.000**	0.000**

II. Antibody kinetics estimation based on Serosolver: Model estimates and sample size by age group, location, and pathogen. Models run with 5 chains and 700k iterations per chain.

Study site, age group and pathogen	Antibody boost, 95% credible interval	Waning, 95% credible interval	N individuals (samples per person)
SOUTH AFRICA			
< 5 yo A/H3	5.50 (5.09, 5.87)	.13 (.08, .16)	91 (2)
< 5 yo A/H1	5.86 (5.31, 6.40)	0.09 (0.00, 0.15)	90 (2)
< 5 yo B/Vic	3.87 (3.12, 4.71)	.13 (.03, .21)	90 (2)
< 5 yo B/Yam	4.66 (4.17, 5.14)	.17 (.07, .25)	90 (2)
5-10 yo A/H3	2.90 (2.67, 3.13)	0.08 (0.06, 0.09)	285 (2)
5-10 yo A/H1	2.88 (2.67, 3.09)	0.04 (0.00, 0.07)	285 (2)
5-10 yo B/Vic	2.14 (1.85, 2.45)	0.04 (0.01, 0.07)	285 (2)
5-10 yo B/Yam	3.17 (2.75, 3.83)	0.09 (0.07, 0.11)	285 (2)
18 + A/H3	3.73 (3.52, 3.93)	0.05 (0.04, 0.05)	653 (2)
18 + A/H1	3.62 (3.48, 3.80)	0.05 (0.04, 0.06)	653 (2)

18+ B/Vic	2.77 (2.56, 2.99)	0.05 (0.05, 0.06)	653 (2)
18+ B/Yam	3.13 (2.95, 3.31)	0.06 (0.05, 0.07)	653 (2)
KING COUNTY			
<5 yo A/H3	4.47 (3.84, 5.72)	.16 (.05, .27)	396 (1)
<5 yo A/H1	4.11 (3.72, 4.57)	.21 (.13, .28)	406 (1)
<5 yo B/Vic	3.97 (3.42, 4.61)	.16 (.08, .24)	407 (1)
<5 yo B/Yam	4.47 (4.12, 4.84)	.25 (.18, .32)	412 (1)
<5 yo RSV	4.67 (4.29, 5.09)	0.07 (0.02, 0.12)	434 (1)
<5 yo CoV N	4.77 (4.38, 5.17)	.15 (.01, .36)	422 (1)
<5 yo CoV Spike	4.97 (4.75, 5.21)	0.05 (0.00, 0.10)	429 (1)
<5 yo CoV RBD	4.86 (4.60, 5.11)	0.11 (0.05, 0.19)	437 (1)
<5 yo HCoV 229E	4.73 (3.94, 5.33)	0.15 (0.09, 0,26)	403 (1)
<5 yo HCoV NL63	4.09 (3.85, 4.38)	0.03 (0.00, 0.08)	397 (1)
<5 yo HCoV OC43	4.36 (3.01, 4.86)	0.14 (0.09, 0.19)	334 (1)

< 5 yo HCoV HKU1	4.06 (3.68, 4.46)	0.06 (0.00, 0,13)	419 (1)
18+ yo A/H3	1.88 (1.53, 2.05)	0.01 (0.00, 0.01)	253 (2)
18+ yo A/H1	1.90 (1.57, 2.08)	0.07 (0.05, 0.08)	254 (2)
18+ yo B/Vic	1.65 (1.62, 1.69)	0.01 (0.01, 0.01)	253 (2)
18+ yo B/Yam	1.36 (1.26, 1.55)	0.03 (0.02, 0.06)	254 (2)
18+ yo RSV	2.66 (2.50, 2.72)	0.01 (0.01, 0.01)	253 (2)
18+ yo CoV N	4.02 (3.84, 4.21)	0.08 (0.01, 0.16)	245 (2)
18+ yo CoV Spike	9.35 (8.95, 9.84)	0.02 (0.00, 0.07)	243 (2)
18+ yo CoV RBD	9.99 (9.34, 10.77)	0.08 (0.00, 0.28)	245 (2)
18+ yo HCoV 229E	1.62 (1.14, 2.50)	0.01 (0.01, 0.02)	245 (2)
18+ yo HCoV NL63	1.25 (1.14, 1.42)	0.03 (0.00. 0.09)	244 (2)
18+ yo HCoV OC43	1.23 (1.12, 1.73)	0.01 (0.00, 0.04)	243 (2)
18+ yo HCoV HKU1	2.55 (2.40, 2.80)	0.01 (0.01, 0.02)	246 (2)

III. Pairwise correlations in antibody concentration levels by pathogen and age group using the MSD multiplex assay, King County population. Each panel represents the correlation between antibody levels for two pathogens in the same individual at any of the sampled time points, grouped by age. Darker blue areas represent higher correlations. Note the higher correlations across SARS-CoV-2 antigens, alpha and beta coronavirus antigens, and influenza antigens.

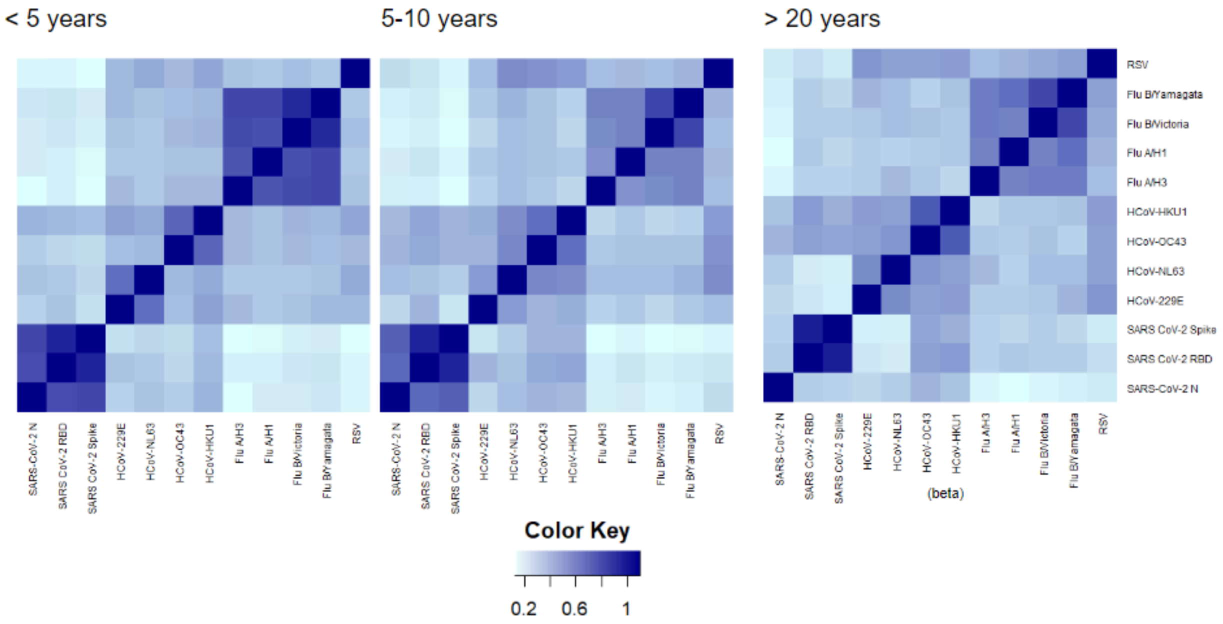

IV. Seasonal incidence of influenza-like Illness (ILI) in Washington State from 2016-2020 (top) and 2019-2023 (bottom), as reported by CDC surveillance.

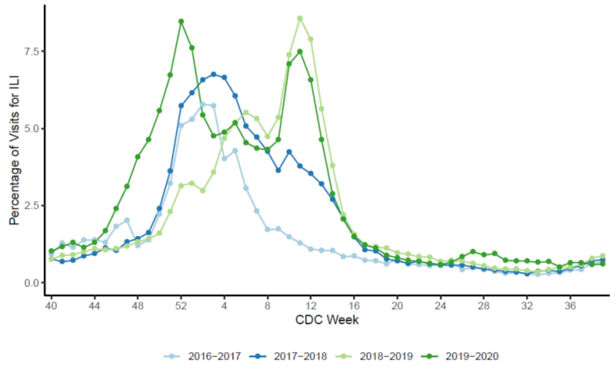

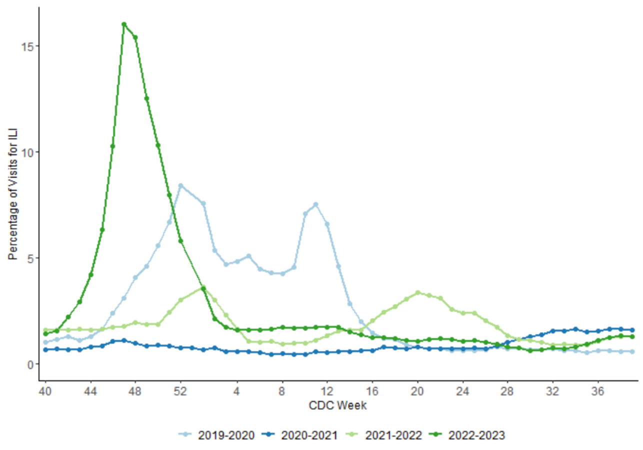

V. South Africa population attack rate observed in empirical data compared to beta distribution prior on attack rates used for Serosolver analyses.

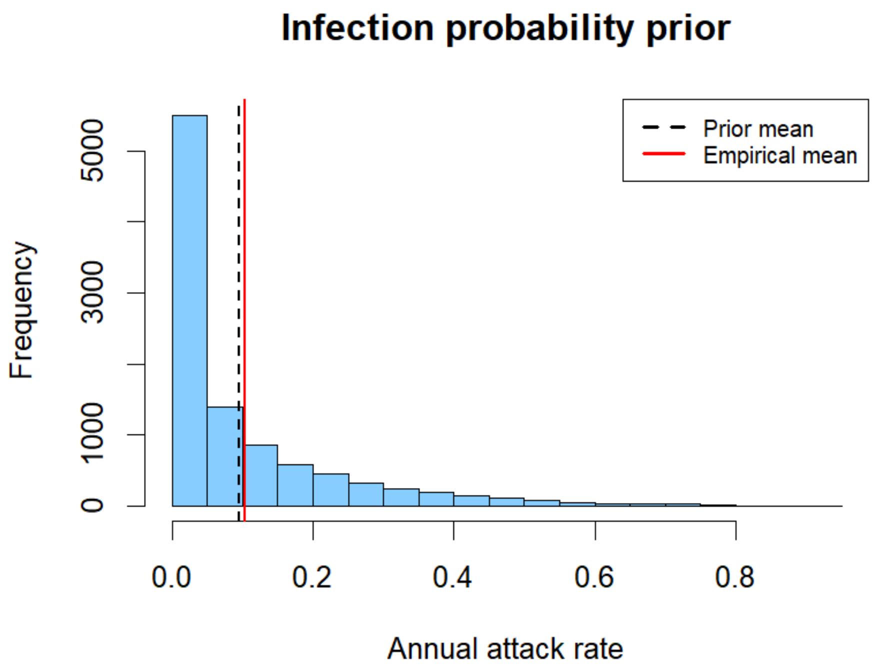

VI. Influenza transmission model parameter table

a. Uniform immunity structure (null model)

Model Parameters	Definition	Source	Value
$1/v$	Duration of immunity (years)	Fixed	4
β_1	Amplitude of seasonal forcing	Fit	.28
1/ϒ	Infectious period (days)	Fixed	2.3
$1/\delta$	Latent period (days)	Fixed	2
Φ	Phase shift	Fit	4.59
β_0	Transmission coefficient	Fit	.53
$\sigma_{1,\ 2,\ 3}$	Proportion of reduced susceptibility via maternal antibodies in months 1,2,3 of infancy	Fixed	.08, .45, .45
h_1, h_2, h_3, h_4	Age-specific hospitalization rates for 0-5, 5-10, 20-64, 65+ age groups	Fit	.12, .09, .06, .10
c_1, c_2	Reduced transmissibility via lockdowns March 2020-March 2021, April 2021-April 2022	Fit	.57, .73

b. Tiered immunity structure model

Model Parameters	Definition	Source	Value
$1/v$	Duration of immunity (years) in individuals > 5 yrs	Fixed	4
r	Change in duration of immunity in children < 5yrs, relative to older individuals (duration of immunity in children is r/v)	Fit	0.49
β_1	Amplitude of seasonal forcing	Fit	.32

1/ϒ	Infectious period (days)	Fixed	2.27
1/δ	Latent period (days)	Fixed	2
Φ	Phase shift	Fit	4.61
β_0	Transmission coefficient	Fit	.51
$\sigma_{1,\ 2,\ 3}$	Proportion of reduced susceptibility via maternal antibodies in months 1,2,3 of infancy	Fixed	.08, .45, .45
$h_{1,}\ h_{2\ ,}\ h_{3,,}\ h_{4,}$	Age-specific hospitalization rates for 0-5, 5-10, 20-64, 65+ age groups	Fit	.13, .20, .08, .08
$c_{1,}\ c_{2\ ,}$	Reduced transmissibility via lockdowns March 2020-March 2021, April 2021-April 2022	Fit	.50, .61
